# Contamination of Medical Charts: An Important Source of Potential Infection in Hospitals

**DOI:** 10.1371/journal.pone.0078512

**Published:** 2014-02-18

**Authors:** Kuo-Hu Chen, Li-Ru Chen, Ying-Kuan Wang

**Affiliations:** 1 Department of Obstetrics and Gynecology, Taipei Tzu-Chi Hospital, The Buddhist Tzu-Chi Medical Foundation, Taipei, Taiwan; 2 School of Medicine, Tzu-Chi University, Hualien, Taiwan; 3 Mackay Memorial Hospital, Taipei, Taiwan; 4 Department of Mechanical Engineering, National Chiao-Tung University, Hsinchu, Taiwan; 5 Department of Nursing, Taipei Tzu-Chi Hospital, The Buddhist Tzu-Chi Medical Foundation, Taipei, Taiwan; Naval Medical Research Unit 6, United States of America

## Abstract

**Objective:**

This prospective study aims to identify and compare the incidence of bacterial contamination of hospital charts and the distribution of species responsible for chart contamination in different units of a tertiary hospital.

**Methods:**

All beds in medical, surgical, pediatric, and obstetric-gynecologic general wards (556) and those in corresponding special units (125) including medical, surgical, pediatric intensive care units (ICUs), the obstetric tocolytic unit and delivery room were surveyed for possible chart contamination. The outer surfaces of included charts were sampled by one experienced investigator with sterile cotton swabs rinsed with normal saline.

**Results:**

For general wards and special units, the overall sampling rates were 81.8% (455/556) and 85.6% (107/125) (p = 0.316); the incidence of chart contamination was 63.5% and 83.2%, respectively (p<0.001). Except for obstetric-gynecologic charts, the incidence was significantly higher in each and in all ICUs than in corresponding wards. Coagulase-negative staphylococci was the most common contaminant in general wards (40.0%) and special units (34.6%) (p>0.05). Special units had a significantly higher incidence of bacterial contamination due to *Staphylococcus aureus* (17.8%), Methicillin-resistant *Staphylococcus aureus* (9.3%), *Streptococcus viridans* (9.4%), *Escherichia coli* (11.2%), *Klebsiella pneumoniae* (7.5%), and *Acinetobacter baumannii* (7.5%). Logistic regression analysis revealed the incidence of chart contamination was 2- to 4-fold higher in special units than in general wards [odds ratios: 1.97–4.00].

**Conclusions:**

Noting that most hospital charts are contaminated, our study confirms that a hospital chart is not only a medical record but also an important source of potential infection. The plastic cover of the medical chart can harbor potential pathogens, thus acting as a vector of bacteria. Additionally, chart contamination is more common in ICUs. These findings highlight the importance of effective hand-washing before and after handling medical charts. However, managers and clinical staff should pay more attention to the issue and may consider some interventions.

## Introduction

Reducing healthcare-associated infection (HAI) remains a critical issue for clinicians and managers in hospitals and healthcare institutions all over the world. Correct hand washing has been proved the most effective way to prevent HAIs [1]–[4]. Based on the WHO guidelines, good hand hygiene can lower the risk of hand transmission of microorganisms [1], [5]. However, it is difficult to examine whether clinical staff conform to the guidelines in daily practice. Worrisomely, previous studies have showed that the compliance with hand hygiene guidelines is low and unsatisfactory among healthcare workers [6]–[9]. Most healthcare personnel do not wash their hands between handling medical charts and touching patients [9]. Despite many attempts to promote or measure the compliance of hand hygiene [5], [8], [10]–[13], adherence remains questionable. In addition, detecting possible vectors of pathologic microorganisms in healthcare institutions is another important step in blocking the transmission or eradicating these pathogens. Although a number of methods, including hand washing, have been used to minimize the occurrence of related infections, there has not been much focus on the source of potential infection in the environment, particularly, the role of hospital medical charts as a possible vector of pathogens.

It was previously shown that stethoscopes, white coats, keyboards, faucets, mobile phones, writing pens, case notes, medical charts, and even wrist watches can be contaminated by environmental or pathologic microorganisms such as methicillin-resistant *Staphylococcus aureus* (MRSA), vancomycin-resistant enterococci (VRE), *Pseudomonas aeruginosa*, and *Klebsiella pneumoniae* [14]–[27]. Such opportunistic or causative pathogens can be found on the surfaces of these personal belongings and facilities within the wards [14], [16], [20], [22], [26]. However, there are few studies on bacterial contamination of hospital medical charts, and two of these reports are a brief report and a letter, respectively [23]–[25]. As pioneers, these pilot studies have been exploratory and were conducted with relatively small sample sizes in selected wards. Furthermore, in some studies, the objects of potential contamination were sampled by means of purposive sampling rather than a general survey, which inevitably affects the results of these studies. In addition, excessively short or long hospitalizations may be major confounders of sampling medical charts, and failure to consider the average hospital stay would confound the results of these studies. In this prospective study, we aimed to identify and compare the incidence of bacterial contamination of hospital charts as well as the distribution of species responsible for chart contamination between different units of a tertiary hospital, while considering the influence of confounders. Using a general survey, all qualified medical, surgical, pediatric, and obstetric and gynecologic (Obs-Gyn) charts were sampled using strict exclusion criteria in order to reach a reliable conclusion.

## Methods

### Study Design and Sample

This prospective study was conducted between January 1, 2010, and December 31, 2010, at a 1,000-bed tertiary hospital in Taipei, Taiwan. The study was approved by the Biosafety Ethics Board of Taipei Tzu-Chi Hospital, The Buddhist Tzu-Chi Medical Foundation. Certain hospital units were excluded from evaluation. Medical charts in the psychiatry, hospice, and burn units were excluded because they were placed in a “discrete” pattern (kept on each bedside table) in contrast to the charts in other general wards and ICUs, where charts were placed in a “central” pattern (kept on a chart rack at the nursing station). Additionally, medical charts in the nursery were excluded because in the nursery of our hospital, the medical staff use case notes without a plastic cover, instead of medical charts. Otherwise, medical charts in general wards including medical, surgical, pediatric, and Obs-Gyn wards as well as charts in corresponding special units including medical, surgical, pediatric intensive care units (MICU, SICU, PICU), and obstetric units (including the tocolytic unit and the delivery room) were surveyed for possible contamination. The sampling time was from 9 a.m. to 11 a.m., immediately following the morning shift. In the study, basic information including hospital stay of the patients and also classification of the beds in general wards and special units were obtained from the department of medical affairs. The data “hospital stay of the patients,” retrieved from an administrative database in the department of medical affairs, only described the duration of hospital stay and were “de-linked” data without any identifiable patient information including the name, ID number, gender, age, occupation, telephone, e-mail, and address. Consequently, we did not have access to the patients, and the written informed consents of the patients (from adults or from kin or caretakers on behalf of the children) were not available.

### Medical Charts

In our hospital, medical charts are handled and recorded mainly by the physicans and nursing staff. Due to different characteristics in different units, it is not clear how many times per day the charts are handled in each unit. We only know that the nurses in general wards and those in ICUs have the same frequency of shifts (eight hours per shift; three shifts per day). Also, the physicians in general wards and those in ICUs have the same frequency of shifts (two shifts per day). All physicians and nurses need to handle the medical charts at least once per shift to finish the medical record. Medical charts in general wards and in ICUs are kept on the chart rack at the nursing station, where the charting is done. All the medical charts throughout the hospital are identical and are replaced every 5 years. Basically, medical charts are not specially wiped down unless there are extra instructions or changes of hospital policy.

### Exclusion Criteria

**Figure 1:**
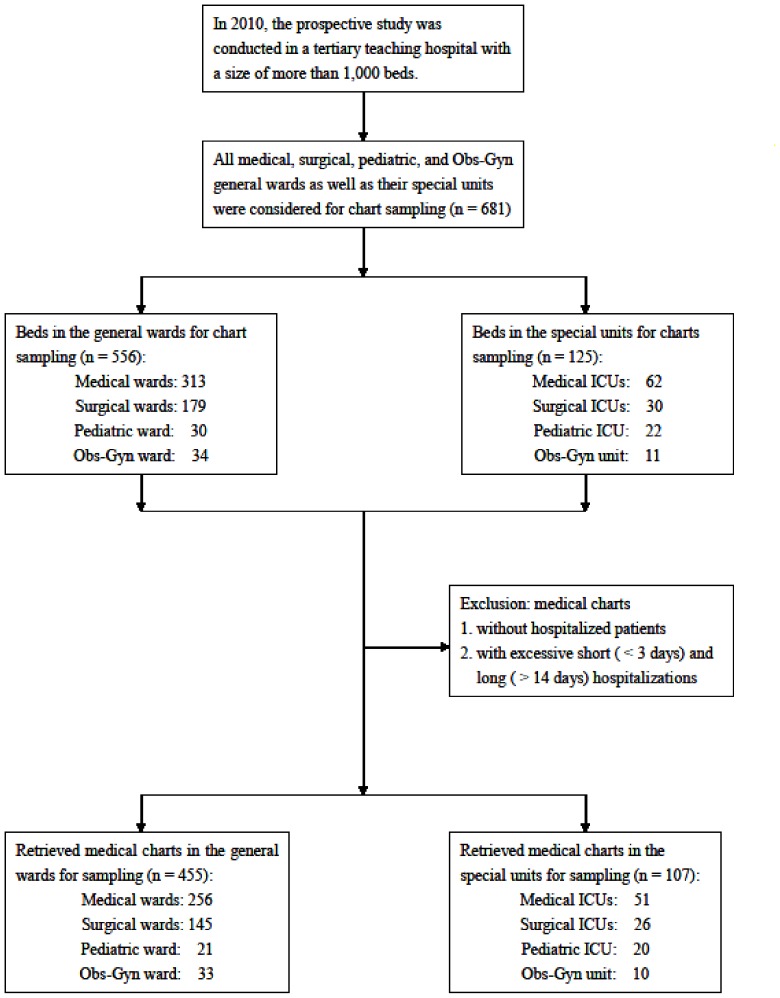
Flow chart for sampling of hospital medical charts. The selection and exclusion of hospital medical charts for sampling to detect possible bacterial contamination.

Considering the differences in frequencies of handling the charts, medical charts of patients who were not hospitalized were excluded in order to avoid selection bias. Furthermore, both of excessively short and long hospitalizations may be major confounders for sampling the medical charts. Since a longer hospital stay may increase the chance of contamination of medical charts, charts of patients who had been in hospital for more than two weeks were excluded. Except for patients in the delivery room with a usually rapid turnover rate, we also excluded patients hospitalized for <3 days. This is because in Taiwan, many minor surgeries or laparoscopic surgeries are performed on patients with a subsequent hospitalization for one or two days based on payment or insurance considerations. In such cases, the physicians and nursing staff often complete all their records including admission, progress and discharge notes at one time and medical charts are handled with low frequency. Otherwise, medical charts that met the inclusion criteria in the general wards and special units were totally collected by means of an ordinary survey rather than being selected according to the investigator's preference (highly selected samples) so as to avoid selection bias (Figure 1). In order to avoid inter-investigator bias and inadequate sampling of the medical charts (measuring bias), only one experienced investigator was responsible for sampling all included charts. Finally, considering the possible effect of time or seasons on organisms, charts in general wards and their corresponding special units (i.e., medical wards vs MICU; surgical wards vs SICU; pediatric wards vs PICU; Obs-Gyn wards vs special units) were sampled in the same month to avoid confounding bias.

### Laboratory Survey

**Figure 2:**
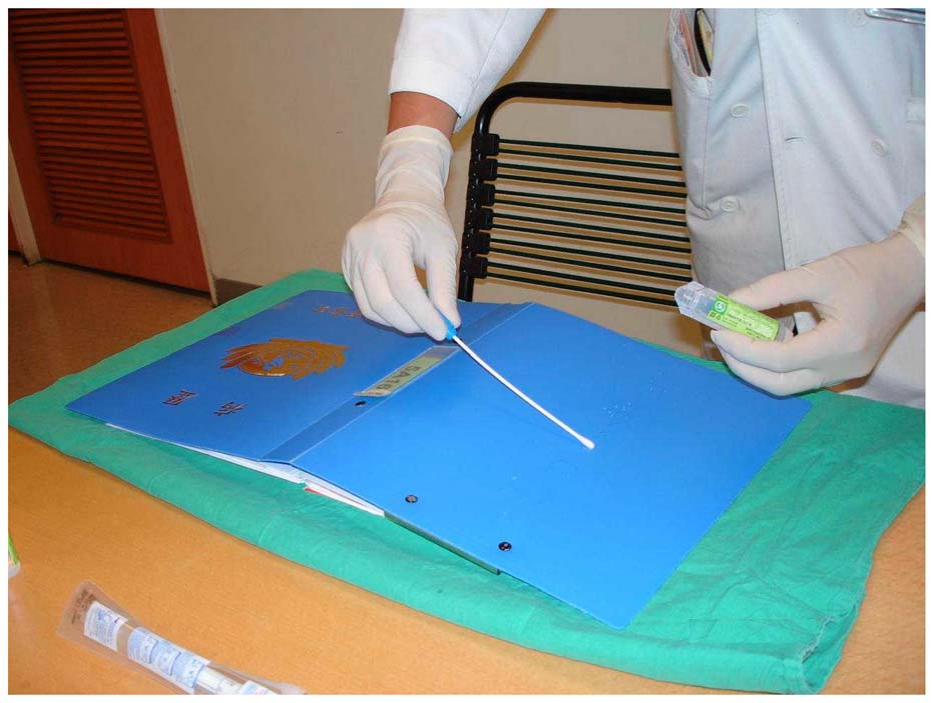
Sampling of a hospital medical chart. The sample was collected from the entire outer surface (plastic cover) of a hospital medical chart with a sterile cotton swab rinsed by normal saline after the experienced investigator had worn sterile gloves.

Samples were collected from the entire outer surfaces (plastic covers) of the hospital medical charts with sterile cotton swabs rinsed with normal saline by an experienced investigator wearing sterile gloves (Figure 2). Prior to transportation, each sampled swab was immediately placed into a special sterile container without spillage or contamination of the sample so as to ensure the accuracy and safety of this study. The swabs along with their containers were then rapidly transferred to the department of laboratory medicine to check the incidence of chart contamination and the bacterial species responsible for said contamination. Cultures were performed according to standard methods used in the hospital [25]. After transportation, each swab was immediately inoculated into a tripticase soy broth and incubated aerobically for 48 hours, then subcultured in a biplate medium composed of sheep blood agar and eosin-methylene blue agar. The identification was carried out using standard microbiological and biochemical laboratory techniques. Cultured organisms were identified using automated methods. If the culture yielded *S. aureus*, the presentation of MRSA was further confirmed by antibiotic susceptibility testing using the disk diffusion technique.

### Outcome Measures

The outcome measures included the overall incidence of bacterial contamination found on hospital medical charts in all general wards and special units, the differences in incidence of bacterial contamination found on medical charts between medical, surgical, pediatric, Obs-Gyn general wards and their corresponding special units, the species of Gram-positive and Gram-negative bacteria on the contaminated medical charts in all general wards and special units, and the differences in distribution of bacterial species on the contaminated medical charts between medical, surgical, pediatric, Obs-Gyn general wards and their corresponding special units.

### Data analysis

Data were collected and analyzed using the SPSS statistical software package (Version 16.0, SPSS Inc., Chicago, IL, USA). The statistics we used in this study included descriptive statistics, the chi-square (X^2^) test, the Fisher's exact test (for expected numbers <5) and the t test to compare differences in the characteristics and the results of the medical charts retrieved for sampling. Further analysis was performed using logistic regression to estimate the odds ratios (OR) and 95% confidence intervals (CI) of the incidence of chart contamination in special units when compared to general wards (the reference group).

## Results

Table 1 summarizes the characteristics and results of the medical charts retrieved for sampling in the study. In this study, we evaluated a total of 681 charts comprising 556 charts of patients in the general wards and 125 charts of patients in the special units,.The 681 beds included 313 medical, 179 surgical, 30 pediatric, and 34 Obs-Gyn beds in the general wards, as well as 62 medical, 30 surgical, 22 pediatric ICU beds, and 11 Obs-Gyn delivery or tocolytic beds. After excluding the beds that did not meet the inclusion criteria, we enrolled 455 beds in the general wards and 107 beds in the special units for chart sampling. In the general wards and special units, the sampling rates of medical charts were 81.8% (455/556) and 85.6% (107/125) for total beds (p = 0.316), 81.8% and 82.3% for medical beds (p = 0.930), 81.0% and 86.7% for surgical beds (p = 0.611), 70.0% and 90.9% for pediatric beds (p = 0.092), and 97.1% and 90.9% for Obs-Gyn beds (p = 0.433), respectively. Of the medical, surgical, pediatric, and Obs-Gyn beds investigated, we found no significant differences in the sampling rates of medical charts between each or between total general wards and special units.

**Table 1:** Characteristics and results of the medical charts retrieved for sampling in the study.

	General Wards		Special Units^1^		
Characteristic					p value
**Sampling rate of medical charts (%)**					
Total	81.8	(455/556)	85.6	(107/125)	0.316
Medical	81.8	(256/313)	82.3	(51/62)	0.930
Surgical	81.0	(145/179)	86.7	(26/30)	0.611
Pediatric	70.0	(21/30)	90.9	(20/22)	0.092
Obs/Gyn	97.1	(33/34)	90.9	(10/11)	0.433
**Average hospital stay** 2 **(days)**					
Total	7.89		7.50		0.099
Medical	9.16		9.31		0.476
Surgical	6.80		7.12		0.292
Pediatric	5.52		5.80		0.529
Obs/Gyn	4.36		2.70		<0.001***
**Contamination rate of charts (%)**					
Total	63.5	(289/455)	83.2	(89/107)	<0.001***
Medical	66.0	(169/256)	86.3	(44/51)	0.004**
Surgical	63.4	(92/145)	84.6	(22/26)	0.042*
Pediatric	52.4	(11/21)	90.0	(18/20)	0.015*
Obs/Gyn	51.5	(17/33)	50.0	(5/10)	0.933

*P<0.05;

**P<0.01;

***P<0.001: chi-square test for percentages of categorical factors of expected numbers >5, Fisher's exact test for percentages of categorical factors of expected numbers <5, student t test for average hospital stay.

1 Including medical, surgical, pediatric intensive care unit and obstetric special units (the tocolytic unit and delivery room).

2 Average hospital stay of the patients corresponding to the medical charts retrieved for sampling.

Of the medical charts retrieved for sampling, the average hospital stay of the patients was 7.89 days in general wards and 7.50 days in special units (p = 0.099); 9.16 days in medical wards compared with 9.31 days in the MICU (p = 0.476); 6.80 days in surgical wards verses 7.12 days in the SICU (p = 0.292); 5.52 days in pediatric wards compared with 5.80 days in the PICU (p = 0.529); and 4.36 days in Obs-Gyn wards vs 2.70 days in the Obs-Gyn special units (p<0.001). Except for patients admitted to Obs-Gyn wards, there were no significant differences in the average length of hospital stay between the patients in medical, surgical, or pediatric wards and those in their corresponding ICUs.

**Figure 3:**
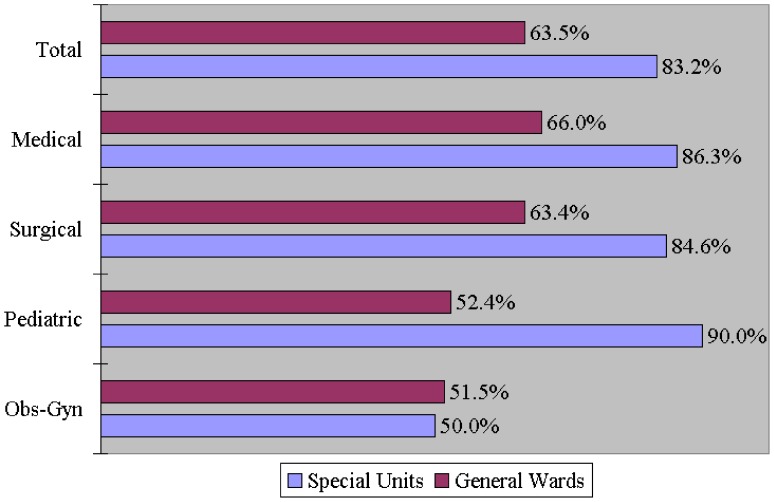
A comparison of the incidence of bacterial contamination on sampled medical charts between general wards and special units.

The contamination rates of medical charts selected for sampling were 63.5% in all general wards and 83.2% in all ICUs (p<0.001); 66.0% in medical wards and 86.3% in the MICU (p = 0.004); 63.4% in surgical wards and 84.6% in the SICU (p = 0.042); 52.4% in pediatric wards and 90.0% in the PICU (p = 0.015); 51.5% in Obs-Gyn wards and 50.0% in the Obs-Gyn special units (p = 0.933) (Table 1). With the exception of charts in Obs-Gyn- associated wards and special units, the incidence rates of chart contamination were significantly higher in each ICU than in the corresponding general ward. On the whole, the incidence of chart contamination was significantly higher in all ICUs than in all general wards (Figure 3).

Table 2 presents a comparison of cultured bacteria from the contaminated medical charts in the study. Of the included medical charts in general wards (N = 455) and those in special units (N = 107), the predominant cultured bacterial species was coagulase-negative staphylococci (CoNS) (n = 182; 40.0% in general wards and n = 37; 34.6% in special units; p>0.05). With the exception of CoNS, the incidence of chart contamination by Gram-positive bacteria, including *Staphylococcus aureus* (n = 19; 17.8%), *Enterococcus faecalis*, *Streptococcus viridans* (n = 10; 9.4%), *Corynebacterium* spp. (n = 11; 10.3%), and *Bacillus* spp. (n = 10; 9.4%), as well as the incidence of chart contamination by Gram-negative bacteria, including *Sphingomonas paucimobilis*, *Pseudomonas aeruginosa*, *Escherichia coli* (n = 12; 11.2%), *Klebsiella pneumoniae* (n = 8; 7.5%), *Pantoea* spp., and *Acinetobacter baumannii* (n = 8; 7.5%), was significantly higher in special units when compared with those in general wards. In addition, the incidence of chart contamination by MRSA was significantly higher in special units (9.3%) when compared with that in general wards (4.0%). Using logistic regression analysis, we showed that the odds ratios of bacterial contamination in special units ranged from 1.97 [95% CI: 1.10–3.53] for *S. aureus* contamination to 4.00 [95% CI: 1.51–10.64] for *K. pneumoniae* contamination, and the odds ratio of bacterial contamination by MRSA was 2.50 [95% CI: 1.12–5.59] in special units when compared with general wards.

**Table 2:** A comparison of cultured bacteria from the contaminated medical charts in the study.

	General Wards		Special Units^1^			
	(N = 455)		(N = 107)			
Bacteria	n	%	n	%	OR	[95% CI]
**Gram positive**						
Coagulase-negative staphylococci	182	40.0	37	34.6	0.79	[0.51–1.23]
*Staphylococcus aureus*	45	9.9	19	17.8	1.97*	[1.10–3.53]
MRSA^2^	18	4.0	10	9.3	2.50*	[1.12–5.59]
*Enterococcus faecalis*	11	2.4	7	6.5	2.82*	[1.07–7.47]
*Streptococcus viridans*	20	4.4	10	9.4	2.24*	[1.02–4.94]
*Corynebacterium* spp.	23	5.1	11	10.3	2.15*	[1.01–4.56]
*Bacillus* spp.	17	3.7	10	9.4	2.66*	[1.18–5.98]
Others	15	3.3	9	8.4	2.69*	[1.15–6.33]
**Gram negative**						
*Sphingomonas paucimobilis*	9	2.0	6	5.6	2.94*	[1.02–8.46]
*Pseudomonas aeruginosa*	6	1.3	5	4.7	3.67*	[1.10–12.25]
*Escherichia coli*	23	5.1	12	11.2	2.37*	[1.14–4.94]
*Klebsiella pneumoniae*	9	2.0	8	7.5	4.00**	[1.51–10.64]
*Pantoea* spp.	9	2.0	7	6.5	3.47*	[1.26–9.54]
*Acinetobacter baumannii*	14	3.1	8	7.5	2.55*	[1.04–6.23]
Others	8	1.8	6	5.6	3.32*	[1.13–9.78]

*P<0.05;

**P<0.01: Odds ratios (OR) and 95% Confidence Intervals (95% CI) are calculated by logistic regression and expressed as compared to the reference group (general wards).

1 Including medical, surgical, pediatric intensive care unit and obstetric special units (the tocolytic unit and delivery room).

2 Methicillin-resistant *Staphylococcus aureus*.

## Discussion

The results of this study revealed that most medical charts were contaminated by bacteria (63.5% in general wards and 83.2% in special units). In addition, medical charts in medical, surgical, and pediatric ICUs were more likely to be contaminated than those in each corresponding general ward (p<0.05), with the exception of Obs-Gyn units. Our data suggest that the medical chart is indeed a possible vector of bacteria and also a potential source of infection. This is particularly true of medical charts in the ICUs. The plastic covers of medical charts can harbor potential pathogens. In addition to CoNS, other causative and opportunistic pathogens were found on the surfaces of medical charts and the risk of chart contamination by these pathogens was 2- to 4-fold [OR: 1.97–4.00] higher in special units than in general wards. The incidence of chart contamination by MRSA was also significantly higher in special units (9.3%) when compared with that in general wards (4.0%). Logistic regression analysis showed that the odds ratio of bacterial contamination by MRSA was 2.50 [95% CI: 1.12–5.59] in special units when compared with general wards. In this study, the incidence of MRSA colonization in special units (9.3%) was even higher than that (6.8%) reported previously [20]. Increased chart contamination by MRSA, one of the most common nosocomial pathogens, is a serious problem in ICUs. Chart contamination by other pathogens is also more common, and probably increases the risk of nosocomial infection.

A number of organisms, such as CoNS, *Corynebacterium* spp., and *Bacillus* spp., are common skin floras, and are considered relatively avirulent although they can be pathogenic in certain populations such as immunocompromised persons with prosthetic devices, intravascular catheters, or other implanted devices. In fact, CoNS has become one of the most common nosocomial pathogens in the hospital setting, and most species are multidrug- resistant [28]. We reanalyzed our data after excluding chart contamination by CoNS, *Corynebacterium* spp., and *Bacillus* spp., and focused on chart contamination by other organisms that are regarded as pathogenic. Our data showed that the contamination rates of medical charts selected for sampling were 32.7% (149/455) in all general wards and 65.4% (70/107) in all special units. Our re-analysis data indicate that chart contamination is still common even after exclusion of those deemed environmental flora.

In order to explain the possible mechanisms underlying our data, we suggest that our results are related to the sources and the frequencies of “contact” within the wards or special units of the hospital. Medical charts are handled by physicians, nurses, and other medical staff while recording, looking-up and handing over to the next shifts. The charts are placed in nursing stations, in medical record rooms, or on the beds to be sent to examination rooms, operation rooms, or therapeutic rooms, and therefore are prone to bacterial contamination. In the ICUs, the use and manipulation of endotracheal and gastrointestinal tubes, which are possible sources of contaminants, may result in excessive bacteria transfer, contributing to a higher incidence of contamination of medical charts. This is not the case in Obs-Gyn special units, including delivery and tocolytic units, where endotracheal and gastrointestinal tubes are used less frequently. The lower rate of chart colonization (50.0%) on obstetric special units may also be related to the characteristics of the pregnant women, who are usually a younger, generally healthier patient population with less antibiotic exposure. In summary, strict adherence to appropriate hand–hygiene measures and avoiding the unnecessary use of indwelling catheters and manipulation of invasive devices may be relatively simple means of reducing the transmission or spread of bacteria.

The study herein has a number of strengths, including its relatively large sample size and a high sampling rate of hospital charts. Overall, a total of 562 charts were sampled and the total sampling rate exceeded 80% (81.8% for general wards and 85.6% for special units). Our results are therefore robust due to minimization of possible errors that originate from the sampling process. To the best of our knowledge, this is the largest study of its kind to investigate the contamination of the hospital charts. Additionally, all bias resulting from the samples, from the investigator, from the sampling and measuring process were minimized as much as possible by the methods used. The patients in the two groups (general wards and special units) were comparable in terms of their length of hospital stay because of these exclusions of patients with very short or longer stays. Therefore, any differences in colonization rates were not related to the duration of hospitalization. The fact that there were notable differences in average hospital stay but unremarkable differences in chart contamination between Obs-Gyn general wards and special units may be explained by the special characteristics of pregnant patients in Obs-Gyn special units such as the delivery room, in which there is usually a rapid turnover of hospitalization and less infectious sources. Nonetheless, the percentage of Obs-Gyn beds in total beds was low (45/681 = 6.61%), and this deviation should not affect the main result of the study.

Our study has some limitations. First, we collected medical charts for sampling in a large hospital. Thus, generalization of the conclusions to smaller hospitals, local clinics, or other healthcare institutions like nursing homes should be made with caution. Second, some characteristics of the patients whose charts were sampled, including gender and age, were not considered in our study, and could affect the results. As a cross-sectional study, the last limitation concerns some uncontrolled factors varying with time and the change of medical policies or guidelines. A longer follow-up investigation could overcome this problem, and provide more precise results.

In conclusion, the incidence of contaminated medical charts is higher in the special units (medical, surgical, and pediatric ICUs) than in each of the corresponding general wards. Based on the finding that most hospital charts are contaminated by bacteria, our study confirms that a hospital chart is indeed not only a medical record but also an important source of potential infection. Hospital charts, together with stethoscopes, white coats, faucets, and keyboards, have the potential to act as vectors of bacteria. This fact highlights once again the importance of effective hand washing before and after handling medical charts, entering casenotes [20], touching patients, and performing procedures, since effective hand washing is the best way to block the transmission of pathogens from vectors to vectors, and from vectors to hosts [1]–[4], [29]. Nevertheless, managers and clinical staff in healthcare institutions should pay more attention to the issue of chart contamination and may consider some interventions in response to the problem, in order to reduce possible HAIs and to promote quality of medical care and patient safety. For example, periodic disinfection of hospital charts and medical equipment with alcohol seems a reasonable approach to eradicate pathogens and lower transmission rates. In a future study, we plan to investigate the effects of regular cleaning by an intervention of wiping down the surface of the chart. It will also be interesting to see if modification of traditional plastic covers with anti-bacteria materials, like nano-materials, can prevent the adherence of bacteria to the outer surface of the medical chart. Alternatively, the use of electronic medical records instead of hard medical charts may theoretically decrease the opportunity of contact. By doing so, clinical staff could avoid direct contact with hard medical records as the vectors of pathogens. Additionally, clinical staff could view the medical information of the patients on-line without the use of medical charts, although some contact with the interface (keyboards or screens) is still inevitable. A detailed discussion of these future attempts is beyond the scope of our study, but we believe that further efforts could be made to explore the relationships between contaminated medical charts and HAI, as well as all feasible attempts in the future.
